# Spatio‐temporal digital markers of behavioural and psychological symptoms to support clinical dementia care

**DOI:** 10.1002/alz70858_100300

**Published:** 2025-12-25

**Authors:** Leia Shum, Yasser Karam, Zain Hasan, Shehroz Khan, Andrea Iaboni

**Affiliations:** ^1^ KITE Research Institute, Toronto Rehabilitation Institute ‐ University Health Network, Toronto, ON, Canada; ^2^ University of Toronto, Toronto, ON, Canada

## Abstract

**Background:**

Real‐time location systems (RTLS) are increasingly used within nursing homes and seniors' residential settings, primarily as safety and nurse call systems. Beyond location monitoring, RTLS collects rich information about movement over time, which can be processed to derive clinical insight into resident behaviours and health, as well as measure impact in interventions. The Space‐Time Indices for Clinical Support (STICS) project aims to derive clinical markers and phenotypes of behavioural health in people with dementia to support longitudinal monitoring. This presentation will provide an overview of our work on two behavioural health markers: motor agitation and rest‐activity rhythms.

**Methods:**

In the STICS pilot, location data was collected from a clinical RTLS system installed on a 20‐bed secure inpatient dementia care unit. Weekly health and behavioural assessments supplemented and provided clinical labels for machine learning models. RTLS data was used to build motor agitation detection models based on shift‐by‐shift Pittsburgh Agitation Scale scores and generate six rest‐activity ‘profiles’ using hierarchical clustering methods.

**Results:**

47 people with dementia participated in the study, with a mean of 7 weeks of location data per person. Participants were 45% female, had a mean MMSE of 5/30 (range 0‐23), and a mean baseline NPI of 39 (range 0‐110). 15 participants used gait aids and 10 of 13 wheelchair users could self‐propel. Models distinguished motor agitation from normal motor activities with a best AUROC of 0.81. SHAP explainability analysis determined that 17 of the top 20 model features were RTLS‐based, with movement speed and total distance being key predictors. Using unsupervised deep learning, six digital phenotypes for rest‐activity were identified of which two had well‐regulated circadian rhythms. The remainder included a cluster with high night activity, one with a high degree of day‐to‐day instability, one with a high time in bed over the day and night, and one marked by severe rhythm disturbance.

**Conclusions:**

RTLS systems are a low‐effort method to objectively track changes in resident movements and behaviours longitudinally. We demonstrated that RTLS‐derived digital markers can describe behavioural symptoms in dementia, although further validation in long‐term care settings is needed.

